# Last-gen nostalgia: a lighthearted rant and reflection on genome sequencing culture

**DOI:** 10.3389/fgene.2014.00146

**Published:** 2014-05-22

**Authors:** David Roy Smith

**Affiliations:** Department of Biology, University of Western OntarioLondon, ON, Canada

**Keywords:** bioinformatics software, electropherogram, next-generation sequencing, nuclear genome assembly, Sanger sequencing

I sometimes see them in my dreams. The colorful peaks and troughs, the sharp, crisp waves spread across my computer screen, the rolling nitrogenous mountains, each with its own nucleotide sitting solidly on the summit. I'm talking about electropherograms, of course. Remember them? Those beautiful but oh so “old-gen” bioinformatics data generated from automated Sanger sequencing machines, such as the Applied Biosystems 370—the geriatric of genome sequencers. Don't laugh. It was these capillary-based electrophoretic technologies that gave us the draft human genome sequence (Lander et al., 2001) and the genome maps of many other model organisms, from the bacterium *Haemophilus influenza* to the yeast *Saccharomyces cerevisiae* to the multicellular green alga *Volvox carteri* (Fleischmann et al., 1995; Goffeau et al., 1996; Prochnik et al., 2010).

As a grad student, I spent countless hours pruning, editing, assembling, and occasionally oohing and awing over Sanger sequences (Sanger et al., 1977; Smith et al., 1986; Prober et al., 1987). These 800-nucleotide genetic snippets intrigued, inspired, and motivated me. They contained just enough data to pique my interests—a novel exon, strange repeat, or foreign gene—and always left me craving a bit more: one additional sequencing read to extend that PCR product, find that stop codon, or join those lonely contigs. Usually, it would take weeks or months to get that extra read, and when it arrived I would savor the experience, exploring and analyzing it like a new book from a favorite author. After I devoured the data, I would say to myself, “If only I could get my hands on a great number of sequencing reads from my organism of interest then all of my genomic woes would be over.” Naively, I believed that the more sequencing data I had, the more productive I would be. Be careful what you wish for from the genome gods. The onslaught of next-generation sequencing (NGS) technologies (Metzker, 2010; Koboldt et al., 2013) and the access to previously unfathomable amounts of genomic data have made me dizzy, disillusioned, and anything but efficient.

Like the proverbial boiling frog, my mind is gradually overheating from an accumulation of NGS reads (Liu et al., 2012). It's a paired-end nightmare, a SOLiD pain in the neck, and a massively parallel migraine. All this HiSeq and MiSeq is clogging-up my internal drive and externals disks. I've taken vacations and returned home only to find that my Illumina reads still haven't finished downloading. I can't move or backup a FASTQ file without needing a coffee break. Last month it got so bad that I tried calling 911 on my 454. I'm certain that I would have had two *Nature* papers by now if it weren't for that pestering computer cursor that keeps spinning around and around, reminding me of my small memory and pitiful processing power.

With all this NGS information, what have I gained (apart from being a chronic user of SEQanswers.com)? Well, I'm a co-investigator of a half a dozen, highly fragmented nuclear genome assemblies for various green algae, with no genome papers anywhere in sight. And don't get me started on the number of transcriptome projects waiting to be written up. What's worse is that I'm still sending more samples for sequencing. It's become my default setting: when in doubt, sequence. If a colleague drops by my office and says, “Smitty, you interested in milkweeds?” My first response is, “You betcha. Let's send some for sequencing?” Student asks: “Professor Smith, do you have any ideas for my honors thesis?” “Hmmm,” I say, “how about we sequence another green alga.” Grant money left over, what do I do? You guessed it: two for one RNA-seq at the campus sequencing facility. And if the data come back contaminated or the quality is poor? Easy, I sequence more! It's gotten to the point where I should begin my conference presentations with, “Hello, my name is David and I'm a NGS addict.”

There are some positives to being NGS obsessed. I'm constantly testing and learning the newest bioinformatics software and genome assembly programs. I know all of the hippest genome slang and genetic acronyms. I have learned more than I ever wanted to about Linux, Unix, and Perl, although, as my students regularly point out, I'm still a hack in all three of those areas. I love that I can go to the Sequence Read Archive at the National Centre for Biotechnology Information (Leinonen et al., 2011) (I visit the site incessantly) and in seconds access endless amounts of raw genomic and transcriptomic data from some of the coolest and most bizarre species on earth, and then use these data to mine genes for phylogenetic and other comparative analyses. I'm also an organelle genome junkie, and NGS techniques have made it quick and easy for me to sequence or data mine complete mitochondrial and plastid DNAs from a diversity of interesting taxa throughout the eukaryotic tree of life (Smith, 2012).

Sequencing nuclear DNAs has been a different story. Even with huge datasets, state-of-the-art assembly programs, and intricate annotation pipelines, I'm incapable of producing decent nuclear genome assemblies. It doesn't help that the species I choose to investigate are poorly studied and poorly sequenced. For researchers investigating organisms for which high-quality nuclear genome assemblies already exist (i.e., assemblies based on Sanger sequencing), the payoffs of NGS have been great (Koboldt et al., 2013). Perhaps as sequencing technologies improve, personal computing power increases, and bioinformatics software become more user friendly, it will soon be easier for small labs to assemble publication-quality nuclear genomes of non-model taxa. For now, however, the promises of NGS have, at least for me, not lived up to their hype and often resulted in disappointment, frustration, and a loss of perspective.

Don't get me wrong, NGS has revolutionized, accelerated, and, in many ways, simplified scientific research. Moreover, new (and soon to come) long-read technologies will alleviate many of the current limitations of NGS (English et al., 2012), such as the absence of a reference genome map. But no matter how long sequencing reads get, NGS will probably never be the panacea of genetics that some claim it to be (Koboldt et al., 2013).

I was taught to approach research with specific hypotheses and questions in mind. In the good ol' Sanger days it was questions that drove me toward the sequencing data. But now it's the NGS data that drive my questions. I recently sequenced the transcriptome of a saltwater *Chlamydomonas* alga and have been knocking my head against the laboratory door asking, “What is the best way to market, package, and publish these data?” I'm trapped in a cycle where hypothesis testing is a postscript to senseless sequencing (Smith, 2013).

As we move toward a world with infinite amounts nucleotide sequence information, beyond bench-top sequencers and hundred-dollar genomes, let's take a moment to remember a simpler time, when staring at a string of nucleotides on a screen was special, worthy of celebration, and something to give us pause. When too much data were the least of our worries, and too little was what kept us creative. When the goal was not to amass but to understand genetic data.

I have a colleague on the inside—works at a big genome-sequencing centre in California. We had lunch recently and during one of my rants he stopped me and said, “Dave, take it easy, we still got them, a whole factory floor of AB3730xl Sanger sequencers!” Later that month, for old-time's sake, I sent him a few PCR products, which were kicking around the lab, and, sure enough, 2 weeks later three electropherograms arrived in my Inbox, like long lost friends. Anyway, for all those Sanger sequencing geeks out there, caught in a next-gen maze of short reads and long headaches, this one's for you.

## Conflict of interest statement

The author declares that the research was conducted in the absence of any commercial or financial relationships that could be construed as a potential conflict of interest.
